# The effects of growth hormone on therapy resistance in cancer

**DOI:** 10.20517/cdr.2019.27

**Published:** 2019-09-19

**Authors:** Reetobrata Basu, John J. Kopchick

**Affiliations:** ^1^Ohio University Heritage College of Osteopathic Medicine (OU-HCOM), Ohio University, Athens, OH 45701, USA.; ^2^Edison Biotechnology Institute, Ohio University, Athens, OH 45701, USA.

**Keywords:** Growth hormone receptor, growth hormone, therapy resistance, chemoresistance

## Abstract

Pituitary derived and peripherally produced growth hormone (GH) is a crucial mediator of longitudinal growth, organ development, metabolic regulation with tissue specific, sex specific, and age-dependent effects. GH and its cognate receptor (GHR) are expressed in several forms of cancer and have been validated as an anti-cancer target through a large body of *in vitro*, *in vivo* and epidemiological analyses. However, the underlying molecular mechanisms of GH action in cancer prognosis and therapeutic response had been sparse until recently. This review assimilates the critical details of GH-GHR mediated therapy resistance across different cancer types, distilling the therapeutic implications based on our current understanding of these effects.

## Introduction

Development of resistance to therapy is one of the toughest challenges in disease management. On one hand, as scientists delve into comprehending the disease towards identifying a cure, another complex challenge is inherently conceived in the development of resistance towards a specific therapy. Cancer accounts for the maximum number of deaths in current times, second only to cardiovascular disease. Almost all our organs are susceptible to neoplastic transformations not only through genetic or epigenetic alterations, but also by complex interactions with the non-cancerous cells in the tumor microenvironment. These transformed cells can collectively progress to a state of malignancy, followed by metastasis, and subsequently death. Currently, the cancer field is equipped with multiple methods of detection as well as multiple modalities of therapy using surgery, radiation, chemotherapy, targeted-therapy, immunotherapy and combinations of these - leading to a 26% drop in cancer death rates in United States since 1991. However, despite this positive improvement in treatment, there were 1,735,350 new cancer cases, 609,640 cancer-related deaths, and a $147.3 billion national expenditure for cancer care in the United States in 2017 (SEER program, National Cancer Institute, www.cancer.gov). For more than a hundred other countries as well as in the global scenario, the statistics are significantly worse. Thus, there is still a “standing order” to decipher and overcome the hurdle of therapy refractoriness in cancer, a multi-factorial process with diverse underlying mechanisms^[1]^. A growing body of research appears to indicate that the growth hormone (GH)/growth hormone receptor (GHR) interaction might provide a valuable clue towards a solution^[2-4]^. In this review we analyzed collective reports of GH action in cancer and attempted to clarify the newly understood role of GH in driving tumoral resistance to different anti-cancer treatments.

## GH-GHR action

GH is a central regulator of tissue and organ development, with anabolic as well as catabolic effects in a tissue-dependent manner^[5]^. Centrally, GH is secreted as a peptide hormone in circulation, from the anterior pituitary somatotroph cells in a pulsatile manner, under direct control of hypothalamic neuronal projections^[6]^. GH secretion is mainly modulated positively by growth hormone releasing hormone (GHRH), ghrelin^[7,8]^, and negatively by somatostatin (SST), free fatty acids, and insulin-like growth factor 1 (IGF1)^[4]^, in addition to catecholamines to a limited extent^[9-12]^. Following release, GH can bind to pre-dimerized GHR on cell surfaces^[13-15]^, activating associated kinases like JAK2 and SRC, to initiate a signaling cascade including but not limited to STATs 1, 3, 5, the PI3K-AKT-mTOR, the Grb10-SOS-RAS-RAF-MEK-MAPK, as well as PLC/PKC/Ca^2+^ pathways, in a tissue and cell-specific manner^[16-19]^. GH-GHR interaction is crucial for longitudinal growth by promoting bone, cartilage, and muscle development, and attaining optimum reproductive capacity^[20]^ by effects mediated directly by GH or via GH-stimulated production of IGF1, the surrogate marker for GH action. Importantly, GH also has a profound role in whole-body metabolic homeostasis by virtue of its critical effects on carbohydrate, lipid, and protein production and turnover^[21-23]^ in liver, adipose tissue (AT), and muscle^[24]^ - the organs expressing highest levels of GHR - as well as in other organs like the kidney, pancreas, brain, heart, skin and immune cell populations^[23]^. GH breaks down lipids^[25,26]^, blocks protein degradation, upregulates gluconeogenesis and protein production^[23]^, increases water retention, and modulates albumin and transthyretin levels throughout the human lifespan^[27]^. In adulthood, following sexual maturity, elevated GH levels lead to insulin resistance, reduced stress resistance, and accelerated aging^[28-30]^. A series of studies spanning several decades in human patients and animal models have highlighted that a congenital disruption of GH action can lead to a protective effect from diabetes, cancer, and aging-associated physiological decline including cognition^[2,31-36]^. Landmark studies on cohorts of human Laron Syndrome (LS) patients with a non-functioning GHR, in Israel by Laron and colleagues^[34,37,38]^ and in Ecuador by Jaime Guevara-Aguerra and colleagues^[33,39,40]^, as well as on LS mouse models of GHR knock-out (GHRKO) produced in our laboratory^[2,3,41,42]^ have established the beneficial effects of congenital resistance to GH action. It is important to note here that the observations in congenital or adult-onset GH deficient (GHD) patients are more disparate with very different underlying mechanisms and implications and are not discussed here. Cohorts of GHD patients in Krk^[43,44]^, Itabaianinha^[45,46]^, Sindh^[47]^, Swiss^[48]^, or African pygmies^[49]^ do share defects in the GH axis but not GH alone and have been reviewed elsewhere^[2,42]^. On the other hand, the condition of GH excess, mostly due to a hypersecreting pituitary adenoma, and known as acromegaly^[50]^, when left untreated in children will lead to a condition of unregulated longitudinal growth resulting in gigantism. However, in most patients acromegaly arise at adulthood and is accompanied by increased risks of stroke^[51]^, significantly higher incidence of benign and malignant colorectal and thyroid neoplasms^[52-55]^, insulin resistance and diabetes, diabetic nephropathy^[56-58]^, and multi-organ failures leading to a significantly reduced lifespan^[59]^. Mouse models of GH excess - mice transgenic for bovine GH (bGH) - corroborate the above human data^[2,42,60-62]^. These pleiotropic effects of GH action positions GH as a truly enigmatic biomolecule and a topic of intense research in human health for the last century. Several reviews cited above elaborately discuss the structure, activation, signal transduction, and metabolic effects of GH in health and disease in different tissues. In this review, we exclusively focus on the unique role of GH-GHR in cancer therapy resistance.

## GH-GHR in cancer

A significant volume of research *in vitro*, *in vivo*, in clinical specimens and retrospective meta-analysis on human patients of GH-excess (acromegaly) and GH-resistance (LS) have established that a paracrine/autocrine GH supports oncogenesis and drives neoplasms towards malignancy, metastasis or relapse in multiple tissues^[2,3,63]^. We refer our readers to a series of relevant reviews by us and colleagues in this regard, compiling the systematic comprehension of the overall and molecular details of how GHR-positive cancer cells exploit the versatile effects of GH action^[2-4,64-69]^. In relevance, the association between GH treatment and cancer incidence in GHD patients remains unclear and widely debated^[70]^. A population-based cohort study of 6,874 patients in France reported elevated risk of bone tumors but no other primary cancers in GH-treated GHD patients^[71]^. Recent reports from the Safety and Appropriateness of Growth Hormone Treatments in Europe (SAGhE), the European cohort study across 23,984 patients in eight European countries indicate a distinct risk of cancer, especially Hodgkin’s lymphoma and meningioma^[72]^, in pediatric GHD patients “with previous history of cancer” and treated with GH in childhood^[73]^. However, multiple subsequent reports did not find a consistently elevated risk of cancer incidence or mortality in GH-treated adult GHD patients^[74-76]^. Another multinational observational study from 1999-2015 on 22,311 GH-treated children from 827 investigative sites in 30 countries called GeNeSIS (Genetics and Neuroendocrinology of Short Stature International Study) also did not find a significantly overall increased risk of cancer mortality or incidence^[77]^. On the other hand, the association between cancer risk in human patients of GH-excess/acromegaly has also been unclear. A confounding factor in this case are variations in IGF1-normalizing medical interventions (surgery, GHR-inhibitors, somatostatin-analogs) the study participants underwent prior to the study^[2]^. However, a number of recent large-scale retrospective meta-analyses reveal a distinctly higher standardized incidence ratio for multiple cancers in these patients^[52,78]^. A single nucleotide polymorphism in GHR, as observed in P495T GHR variant in some ethnic groups, impairs the SOCS-mediated deactivation of an activated GHR, thereby prolonging GH action^[79,80]^. This P495T variant with elevated GH-GHR signaling has been associated with markedly increased incidence of lung cancer in patients^[80,81]^. On the other hand, resistance to cancer has been one of the consistent features of the two cohorts of LS patients in both Israel^[34,63,82,83]^ and Ecuador^[33]^, with multiple studies focusing on the underlying molecular mechanisms^[83-85]^. Mouse models of dysregulated GH action - the bGH and the GHRKO mice - closely recapitulates the oncogenic profiles of the corresponding human patients^[2]^. A number of *in vivo* xenograft studies on bGH, GHRKO, as well as on mice transgenic for a GHR-antagonist (GHA mice) revealed an intrinsic resistance to tumor development and cancer progression due to abrogation of GHR function^[2,3,86]^.

**Figure 1:**
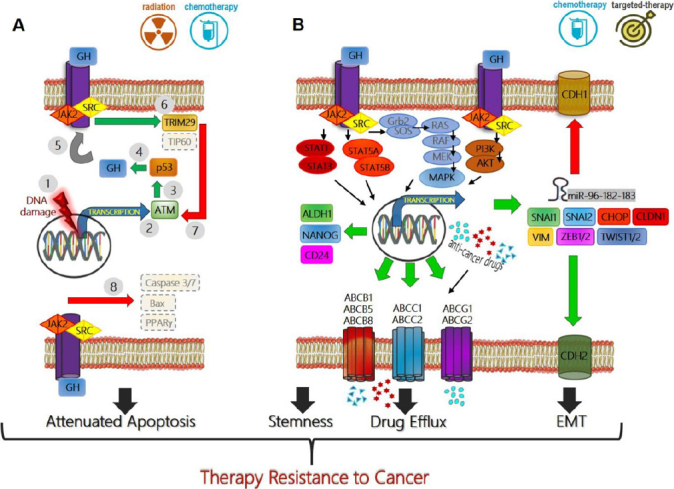
Mechanisms of growth hormone mediated therapy resistance in human cancers: (A) therapeutic interventions (radiation or chemotherapy) which cause DNA damage in tumor cells (1), induce ATM (2) mediated p53 production (3) which directly increases GH production (4). This GH can have an autocrine/paracrine effect on binding to same or neighboring cell surface GH receptors (GHR) (5), initiating a JAK2 and SRC mediated signaling cascades which lead to elevated TRIM29 and decreased Tip60(6), which in turn blocks ATM (7) and decreases p53 via feedback inhibition. GH-GHR interaction also decreases pro-apoptotic molecules (Bax, PPARγ) and suppresses Caspase activation (8) thus allowing escape from cell death and providing resistance to therapy; (B) GH-GHR interaction drives resistance against pharmacologic intervention (chemotherapy or targeted therapy) by upregulating ABC-multidrug efflux pumps, inducing epithelial-to-mesenchymal transition or EMT (elevated mesenchymal transcription factors SNAI1, SNAI2, ZEB1/2, TWIST1/2, CLDN1, VIM, and miRNA cluster 96-182-183, along with decreased CDH1, and increased CDH2) and by inducing markers of stemness like ALDH1, NANOG, and CD24 to effect a phenotype switch. The combination of GH mediated suppression of apoptosis, increased capacity of drug efflux, increased stemness and invasive mesenchymal properties allow therapy resistance, metastasis, and relapse of the tumor. Green arrow indicates upregulation while red arrow indicates downregulation of target gene expression

Several different types of human cancers, including cancers of breast, colon, thyroid, blood, skin, pancreas, liver, endometrium, kidney, lung, stomach, glia, thymus, and brain express GHR^[2]^. In these cancers paracrine/autocrine GH induces oncogenic signaling for classical oncogenic processes like proliferation, migration, invasion, angiogenesis^[87]^, metastasis^[65]^, and avoiding apoptosis^[88]^. IGF1, one of the principal effectors of GH action, is also^[89]^ important in progression of specific transformed cells and in driving therapy resistance in cancer^[90]^. While several reviews have described the effects of GH action in cancer prognosis and progression, the unique role and molecular details of GH-GHR action in promoting the resistance of tumors to therapy has not been reviewed. Here, we exclusively zoom in on this critical aspect of GH signaling in GHR-positive human cancers, which appears to point toward a novel target towards tackling malignant cancer subtypes. The specific reports implicating GH action in mediating therapy resistance in cancer are summarized in Table 1. The underlying molecular mechanisms of GH mediated cancer therapy resistance, based on discussions in the subsequent text, is described in Figure 1.

**Table 1:** List of reports implicating growth hormone in development of therapeutic resistance in human cancers

Type of therapy resistance	Cancer type	Treatment	Mechanistic observations	Ref.
1. Deregulated Apoptosis	Lymphoma	Methyl methanosulfonate (MMS)	GH overexpression → lower Bax, BAD, Caspases-3, -8, -9	[95]
	Colorectal	Nutlin, Etoposide, Radiation	DNA damage → p53 → GH. GH blocks p53 by blocking ATM	[96-98]
	Breast	Doxorubicin	GH induced c-fos	[103,104]
	Breast, Endometrial	Mitomycin-C	Autocrine GH → suppressed DNA damage and reduced apoptosis	[146]
	Endometrial	Doxorubicin, paclitaxel, cisplatin	GH → ERK1/2 and PKC → suppressed Caspase 3/7 activation	[103,105]
	Colon	PPARg ligands	GH → STAT5b → reduced Bax, PPARg	[106]
2. Epithelial-to-mesenchymal transition (EMT)	Breast	Serum withdrawal	GH → p38-MAPK → CHOP	[109,110]
	Breast		GH → elevated miR-96-182-183 cluster → BRMS1L	[196,197]
3. Drug efflux via ABC-transporters	Melanoma	Doxorubicin, paclitaxel, cisplatin, oridonin, vemurafenib	GH → JAK2/STAT5 + SRC → ABCB1, ABCBB5, ABCB8, ABCC1, ABCC2, ABCG1, ABCG2	[132,148]
	Breast	Ruxolitinib	GH → JAK2, AKT, PI3K-AKT, MAPK → drug resistance	[147]
	Breast	Docetaxel	GH → JAK2/STAT5 → ABCG2	[149]
4. Stemness (cancer stem cell)	Breast		MCF7-hGH cells → increased markers of CSCs	[168,169]
	Colon		DLD1-hGH, Caco2-hGH cells → increased ALDH1, NANOG, CD24, CD44, etc	[131]
	Liver		Huh7-hGH, HepG2-hGH cells → suppressed CLDN1, elevated ABCG2, NANOG, etc.	[170]
5. Radiation resistance	Breast, Endometrial	Radiation	GH → increased clonogenicity post-irradiation	[177,185]
	Colorectal	Radiation	GH → reduced DNA damage + increased post-irradiation survival	[186,216]

## GH-GHR in cancer therapy resistance

### Deregulated apoptosis

The mitogenic and anti-apoptotic role of GH as a growth factor is common knowledge. In fact, GH treatment in GH deficient (D) children lowers the apoptosis of CD34+ hematopoietic cell population^[91]^. Chemotherapies like doxorubicin as well as radiation induce cell death by inflicting significant DNA damage selectively in highly proliferative cells in the body, leading to apoptosis or senescence. A critical role in DNA damage repair and cellular commitment to apoptosis, is played by p53 (*TP53* gene), a tumor suppressor protein, and one of the most studied proteins in cancer research^[92,93]^. Functional p53 can lead to senescence by inducing a cell-cycle arrest by activating p21 transcription/translation, which in turn inhibits cell-cycle regulators like cyclin dependent kinase (Cdk) 2 and 4. This leads to reduced pRb phosphorylation which sequesters E2F1 and attenuates transcription of mediators of DNA replication and cell-cycle progression. On the other hand, DNA-damage induces activation of ATM which in turn activates p53 to induce apoptosis by effecting mitochondrial outer membrane permeabilization via transcription of pro-apoptotic proteins like Bad, Bak, Bax, Puma, and Noxa, death receptors like Fas, and apoptosis mediators like Apaf1 and Caspase6. Cancer initiation and progression often is accompanied by loss-of-function p53 mutant protein, while gain-of-function mutations of aberrantly oncogenic p53, especially in hematological malignancies, are also known^[94]^. As early as 2004, GH overexpressing EL4 T-cell lymphomas were reported to have reduced apoptosis when treated with methyl methanosulfonate (MMS) and reduced levels of Bax, BAD, and caspases-3, 8, and 9^[95]^. A series of reports, especially from Melmed and colleagues, described a GH-induced feedback inhibition on p53 following DNA-damage in cells^[96-98]^. They showed that Nutlin-induced DNA damage and induction of the p53/p21 senescent pathway lead to GH expression *in vitro* in rodent primary pituitary cultures, in human pituitary adenoma samples and *in vivo* in C57BL/6 mice^[98]^. Chesnokova *et al*.^[98]^, reported direct p53 binding at sites -1118bp and -680bp upstream of GH transcription start site using ChIP assays, supporting GH as a direct p53-target in senescence, in pituitary adenomas as well as non-pituitary cells^[98]^. This senescence-induced p53-mediated GH production appears to have an autocrine/paracrine as well as intracrine effect^[98-100]^. Interestingly this p53-induced GH subsequently appears to exert an anti-apoptotic effect, part of which is mediated by blocking p53 activation. Chesnokova *et al*.^[96]^, also described this seemingly feedback inhibition of p53 by GH, in an elegant study on development of colonic neoplasms^[96]^. GH was found to suppress p53 as well as the p53/p21 activation in cultured colon cells, in colon tissues *in vivo*, as well as in iPSC-derived intestinal organoids, while upregulating epithelial-to-mesenchymal transition (EMT)^[96]^. Additionally, the GH-deficient Ames’ mice (Prop1^-/-^) as well as the GHRKO mice had higher colonic p53 expression than WT counterparts while APC-deficient Ames’ mouse (APC^min+/-^ Prop1^-/-^) had lower incidence of colonic neoplasms than APC^min+/-^ counterparts^[96]^ highlighting the GH-p53 association. The mechanism of p53 suppression and abetting the oncogenic pathway by GH was recently further clarified to be due to GH induced higher-TRIM29-lower-Tip60 mediated suppression of ATM (Ataxia-Telangiesctasia mutated) activation^[97]^. ATM is induced by DNA-damage and p53 is a primary ATM-target^[93,101]^. A recent study revealed increased DNA damage repair (DDR) in GHRKO mice colon and human non-tumor colon cells (hNCC); whereas etoposide and 500ng/mL GH led to increased transformation of hNCC and increased metastasis in colon tumor xenograft bearing mice^[97]^. Thus locally produced GH, in response to therapy induced DNA damage, clearly foster a “milieu permissive of neoplastic growth”^[96]^ by rescuing cells from committing to senescence^[98]^, protecting them from apoptosis^[96]^, or allowing oncogenic mutations by blocking DDR^[97]^, wherein p53 is a central target of GH-induced oncogenicity and therapy evasion. Podlutsky *et al*.^[102]^ have shown improved DDR capacities and upregulated p53-target genes like Gadd45b and Mdm2 in primary fibroblasts from GH/IGF1 deficient Lewis dwarf rats and dwarf Snell mice^[102]^, further bolster the argument presented here that GH action attenuates p53 action and can be a critical oncogenic factor.

Several other studies provide definitive support to the anti-apoptotic effect of GH action in cancer. Studies by Zatelli and colleagues had reported that chemotherapy induced apoptosis via JNK expression and phosphorylation, was blocked by GH - an effect reversed by GHR-antagonist, pegvisomant, in triple-negative breast cancer (TNBC) cells^[103]^. Their studies also found that in TNBC, GH did induce drug-resistance independent of IGF1, by directly inducing c-fos and suppressing apoptosis^[104]^. In a recent study by the same group, GH was found to confer chemoresistance from doxorubicin, paclitaxel, and cisplatin in human endometrial adenocarcinoma^[105]^. In human endometrial cancer, GH was found to suppress caspase 3/7 activation and appeared to function differentially either through the ERK1/2 or PKC pathways depending upon the drug or the cell line; again pegvisomant was found to reverse the effects^[105]^. Bogazzi *et al*.^[106]^ had proposed another mechanism of the anti-apoptotic effects of GH where it blocks the expression of pro-apoptotic PPARγ and Bax in colon cancer cells^[106]^. This survival advantage of tumors bestowed upon by GH to evade the DNA damaging effects of therapy and avoid apoptosis, were also reported in pancreatic cancer^[107]^, and breast cancer^[108-110]^. Therefore, there appears to be a consensus over the anti-apoptotic effects of GH which is harnessed by the proliferative tumor cells; while the details of molecular events converging to the net effect of escaping cell death are overlapping and still emerging.

### EMT

EMT is a biological process involved in diverse cellular contexts like organ development (type 1 EMT), tissue regeneration/wound healing and organ fibrosis (type 2 EMT), and neoplastic events as observed in tumor cells (type 3 EMT). Several excellent reviews have thoroughly described the versatile aspects of EMT in all the above contexts, including in cancer^[111-115]^. Currently, it is well established that EMT is a critical juncture in the life-cycle of a tumor and a determinant in the subsequent fate in tumor death or survival against therapeutic challenges as well as for subsequent successful metastasis^[113,114,116-118]^. In cancer, a fraction of the highly proliferating tumor cells undergoes EMT, when induced by mutational changes and increased DNA damage as a result of therapy. The process of EMT is abetted by growth factor mediated increase in EMT specific transcription factors like Snail, Slug, Zeb1, Zeb2, Twist1, and others which drive a massive concerted change in gene transcription leading to a “switch” in cellular identity and phenotype from “epithelial” to that of “mesenchymal”. Through an elaborate reprogramming of gene expression and cytoskeletal reorganization, the cells lose their adherens junctions, depolarize and assume spindle-shape, and secrete proteases to breakdown extracellular matrix (ECM) and to activate more potent inducers of EMT like TGFβ^[112]^. This switch in phenotype is accompanied by a convergence of increased invasive properties, increased drug-efflux capacity, acquisition of stem-cell markers^[116]^, and resistance to apoptosis^[111,112]^. In the last few years, the EMT process has been found to be more closely linked to chemoresistant metastasis than metastasis alone. Using elegant EMT lineage tracing models Fischer *et al*.^[119]^ had demonstrated that in primary mammary tumor bearing mice cyclophosphamide (CTX) treatment selected for tumors which underwent EMT (GFP+) to achieve a mesenchymal phenotype compared to in the untreated tumor cells. The same set of GFP+ cells expressed significantly more multidrug efflux transporters (Abcb1a, Abcb1b, ABcc1), cytochrome P450s (CYPs), and aldehyde dehydrogenases (ALDHs)^[119]^ - all mediators of chemoresistance. Intriguingly, the suppression of EMT transcription factors by overexpressing miR200, did not affect the rate of lung metastasis of primary mammary tumors, but proved essential to overcome CTX treatment^[119]^. In multiple recent independent studies Snail, Slug, Zeb1, and Twist1 were all found to specifically orchestrate resistance to both chemotherapy or radiation treatment in ovarian^[120]^, nasopharyngeal^[121]^, gastrointestinal^[122]^ and lung^[123,124]^ cancers. The process of EMT in cancer cells thus appears to be initiated by a therapeutic challenge, catalyzed by favorable growth factors. Several studies have reported that GH is a potent inducer of EMT in tumor and normal cells, directly as well as via secondary effectors like IGF1 and TGFβ (reviewed in^[65]^).

Elaborate studies by us and others have identified a direct association of GH and EMT in GHR expressing human cancers^[2,65]^. A series of previous and ongoing work by Peter Lobie’s group have described the potent cancer driving properties of autocrine/paracrine GH using breast cancer cell line MCF7 and its mutant variants - MCF7-hGH and MCF-MUT, with constitutive expression or absence of transgenic hGH respectively^[109,125]^. His group showed that GH stimulated the expression of EMT transcription factors Snail, Slug, downregulated E-cadherin (CDH1), and upregulated mesenchymal markers Vimentin, N-cadherin (CDH2), as well as matrix metalloproteases (MMPs) preferentially in the MCF7-hGH cells. Increased invasive and migratory potential and apoptosis resistance are hallmark feature of cancer cells undergoing EMT^[115,126,127]^. The MCF7-hGH cells were reported to be significantly resistant to apoptosis, mediated by an autocrine GH-dependent p38-MAPK-induced CHOP^[110]^, along with increased metastasis and anchorage abilities in collagen and 3D-matrices^[109]^ due to autocrine-GH-directed HOXA1 mediated expression of cMyc, Cyclin-D1, and Bcl2 - genes involved in facilitating tumor invasion^[128]^. The above phenomenon in MCF7-human (h)GH cells were also found to be JAK2-dependent and was attenuated by AG490, a JAK2-inhibitor^[129]^. Additionally, the β-catenin homolog γ-catenin (plakoglobin) which are involved in tethering E-cadherins at intracellular adherens junctions, were found to have significantly lower expression in the highly invasive MCF7-hGH cells^[125]^, possibly due to a concomitant upregulation of methyltransferase proteins DNMT3A and DNMT3B, which caused plakoglobin transcription arrest by hyper-methylating CpG islands at exon-1 of plakoglobin gene^[130]^. Blocking SRC kinase or MMP9, but not JAK2, attenuated this invasive phenotype of the MCF7-hGH cells^[125]^. Lobie’s group had also identified autocrine GH-regulated miRNA clusters regulating EMT in MCF7 breast cancer cells and will be discussed in a subsequent section. Autocrine/paracrine GH was further found to turn on EMT cascade in human colorectal cancer cells, where E-cadherin was suppressed with a concomitant increase in mesenchymal proteins Vimentin and FN1, via ERK1/2^[131]^; while exogenously added GH increased Snail and Twist2 and suppressed PTEN activity^[96]^. We had reported an increase in mesenchymal proteins N-cadherin and Vimentin and decrease of E-cadherin following a GH dose-dependence in human melanoma^[132]^. Blocking GH signaling by siRNA-mediated GHR knock-down (GHRKD) reversed the effects^[132]^. Consistent results of GH induced EMT were also observed in GHR-expressing pancreatic ductal adenocarcinoma cells following exogenous GH treatment or GHRKD^[107]^. B2036 was reported to also inhibit GH-induced EMT, tumor invasion and anchorage-independent cell growth *in vivo* in endometrial cancer^[133]^. No direct association between GH-induced EMT and GH-directed chemoresistance has been drawn in the above studies, apparently because the role of EMT in driving chemoresistance independent of metastasis have only come to light in recent years, predating the above studies. Apart from driving a highly invasive and metastatic tumoral phenotype, a re-evaluation of earlier reports of GH, EMT, invasion, and therapy-resistance in cancer, collectively does implicate GH in catalyzing EMT-driven evasion of therapy. Thus, from a therapeutic perspective, it would be valuable to know whether the GH induced EMT is critical for driving metastasis or therapy resistance especially in the context of individual cancer types and remains to be delineated through animal studies.

### Drug efflux (multi-drug efflux pumps/ABC transporters)

A multitude of studies have implicated GH action in tumor cells with intrinsic or acquired resistance to chemotherapy like cisplatin, doxorubicin, paclitaxel, Cyclophosphamide (CTX), mitomycin-C (MMC), and others; yet the involvement of membrane-spanning ATP-hydrolyzing multi-drug efflux pumps or ABC (ATP-binding cassette containing)-transporters were not investigated until recently. The ABC-transporter family has 48 members, classified into seven groups ABC-A through ABC-G based on sequence similarities, and involved in efflux of xenobiotics as well as biological macromolecules like peptides and lipids from the cytoplasmic compartment of the mammalian cell^[134-136]^. Excellent reviews have delved into the mechanism of action, subtypes, molecular functions, substrate specificities, and role in health and disease of ABC-transporters, especially in cancer^[134,137-142]^. Over the last 30 years, numerous studies have established ABC-transporters as a major determinant of failure of antineoplastic chemotherapy. The most studied members of the ABC-family of drug efflux pumps in cancer are ABCB1/MDR1/P-gp (P-glycoprotein), ABCC1 and ABCC2 (multi-drug resistance associated proteins 1 and 2; MRP1 and MRP2 respectively), and ABCG2 (breast cancer resistance protein; BCRP)^[143]^. Between them, these major drug-efflux pumps have a wide repertoire of specific as well as overlapping substrates, including antibiotics, antihistamines, analgesics, lipids, neuroleptics, natural products, antihypertensives, HIV drugs, Ca-channel blockers, antivirals, antilipidemics, and anti-cancer drugs. Anti-cancer drugs like doxorubicin, cisplatin, paclitaxel, 5-FU, methotrexate, etoposide, tamoxifen, MMC, vinblastine, and several others are efficiently and rapidly removed from the cytoplasm of tumor cells, following influx, thereby reducing drug retention and efficacy leading to poor prognosis^[134]^. Additionally, especially in melanoma, ABC-transporters play a vital role in drug sequestration inside intracellular vesicles like melanosomes - a process which further protects the tumor from drug action^[144,145]^. Bougen *et al*.^[146]^ have reported that autocrine GH conferred MMC resistance in MCF7, MDA-MB-231, and T47D breast cancer cells in 2D and 3D culture systems and protected them from DNA-damage induced apoptosis^[146]^. Minoia *et al*.^[103]^ and Zatelli *et al*.^[104]^ had reported about GH induced chemoresistance from doxorubicin in MDA-MB-231 and MCF7 breast cancer cells respectively resulting in reduced tumor apoptosis, which was reversed by pegvisomant. A similar effect of GH in protecting endometrial cancer cells from doxorubicin, cisplatin, and paclitaxel treatment was also recently identified by Gentilin *et al*.^[105]^. Further, protection of endocrine-resistant breast cancer from ruxolitinib, a JAK2-inhibitor, was reported to coincide with GHR expression^[147]^. The first clue that GH acts via direct upregulation of ABC-transporter expression in conferring this chemoresistance in tumors came from our study in human melanoma^[2,132,148]^. We observed that doxorubicin, cisplatin, paclitaxel, oridonin, and vemurafenib, in four different human melanoma cell lines, in presence of GH differentially upregulated ABCB1, ABCB5, ABCB8, ABCC1, ABCC2, ABCG1, and ABCG2 multi-drug efflux pump expressions. In fact prolonged GH treatment alone rendered the melanoma cells resistant to chemotherapy, reflected by a two to five fold elevation in the vemurafenib EC50 value^[148]^. GHRKD reversed these effects, increased drug retention, thereby sensitizing the melanoma tumors to low doses of chemotherapy^[148]^. Analysis of murine tumor xenografts in immunocompetent C57BL6/J mouse models of GH excess, e.g., the bovine (b) GH transgenic mouse (bGH), found significantly upregulated ABC-transporters compared to wild-type littermates and verified our *in vitro* observations (unpublished results). Another very recent study in estrogen receptor negative breast cancer cells and in patient-derived nude mice xenografts treated with docetaxel, shRNA mediated silencing of GHR indeed suppressed multi-drug efflux pump ABCG2 and re-sensitized the tumors to the anti-cancer agent^[149]^, thereby providing additional evidence to GHR-mediated induction of drug efflux via upregulated ABC-transporter expression. GHR silencing appeared to concomitantly increase drug-induced apoptosis, and reduced cell viability, migration and invasion properties of the breast cancer cells *in vitro* and *in vivo*^[149]^. Incidentally Wu *et al.*^[150]^ did report that prolactin (PRL), another member of the type 1 cytokine family similar in structure to GH, also conferred docetaxel resistance via ABCG2 upregulation in T47D human breast cancer cells in a JAK2-STAT5 as well as PI3K-MAPK dependent manner. In their study, a putative GAS (gamma interferon activation sequence) motif just 434-bp upstream of the ABCG2 transcription start site, was found to directly bind STAT5 and activate transcription of ABCG2^[150]^. It is highly probable that a direct GH induction of ABC-transporter expression in human cancers proceed through a similar direct binding to GAS elements upstream of ABC-transporters and remains to be verified through future studies. However, the recently identified role of GH action in regulating ABC-transporters is a significant finding, keeping in mind the diverse cellular substrates and established relevance of ABC-transporters in multiple disease states including cancer^[151,152]^, neurological disorders^[153-155]^, obesity^[156]^, cardiovascular diseases^[157,158]^, and more^[159,160]^.

### Cancer stem cells

Cancer stem cells (CSCs) are a sub-population of the tumor bulk characterized by stem-cell like properties of self-renewal, extreme resistance to therapeutic challenges, high degree of invasiveness, heightened survival capacities, and ability to differentiate into aggressive, treatment-resistant tumors^[161,162]^. CSCs resemble quiescent adult stem cells, thus avoiding chemotherapy which primarily targets the highly proliferating population of the tumor bulk and are responsible for tumor dormancy, inevitable recurrence of tumor after an initial successful therapy, and metastasis^[161-163]^. The CSCs overexpress multi-drug efflux pumps (ABCG2, ABCB1, ABCB5), have high propensity to undergo EMT, and are resistant to apoptosis^[161,163]^ - properties catalyzed by GH-GHR interaction on tumors as discussed above. In relevance, agonists of GHRH stimulated self-renewal and survival of cardiac stem cells, via an upregulated GH action^[164]^. Also PRL, had a comparable oncogenic potential in multiple cancer types, exhibited a similar regulatory effect in adult stem cells^[165]^ and in prostate^[166]^ and colorectal CSCs^[167]^.

GH induced phenotypic plasticity resembling CSCs was first observed in autocrine GH expressing breast cancer (MCF7-hGH) cells by Lobie and colleagues^[125,168]^. The promotion of CSC formation via the Wnt/b-catenin pathway proceeds through a necessary downregulation of the epithelial marker E-cadherin^[96]^ and was indeed found to be the case for GH driven acquisition of stemness in breast cancer. Independent research by Lombardi *et al*.^[169]^ showed GHR expression in a subset of normal human breast epithelia that co-expressed stem cell markers but lacked lineage differentiation markers, and could form mammospheres while GHR-negative cells could not^[169]^. Further progesterone stimulation induced GH secretion from normal mammary epithelia, which in turn induced proliferation of GHR-positive mammary stem cells, in an autocrine/paracrine manner^[169]^. The same GHR-positive subsets appeared on 90% of 175 samples of human ductal carcinoma *in situ* (DCIS) lesions which are precursors to invasive breast cancer^[169]^. Additional studies on human colorectal cancer cells DLD-1 and Caco2 as well as their constitutive hGH-expressing variants DLD-1-hGH and Caco2-hGH exhibited more and larger colonosphere formation by the latter group^[131]^. Further in the autocrine GH producing DLD-1-hGH and Caco2-hGH cells, the expression of CSC marker ALDH1 was 2-3-fold higher than DLD-1 and Caco2 set. The RNA expression of CSC markers like CD24, CD44, NANOG, SALL4, and POU5F1 were also exclusively observed in GHR-expressing subsets of the colorectal cancer cells^[131]^. The CSC promoting effect of autocrine GH was further validated in human hepatocellular carcinoma cells Huh7 and HepG2^[170]^. Forced GH expression in stably transfected Huh7-hGH and HepG2-hGH cells induced a JAK2-STAT3 mediated suppression of the tight junction component CLAUDIN1, leading to conferral of CSC properties, including upregulated ABCG2, NANOG, SALL4, and other CSC markers and mediators in these liver cancer cells^[170]^. Collectively, these results do establish a stem-cell promoting property of GH in cancer and warrant further *in vivo* studies in GHR-overexpressing cancers like melanoma, thwarted by drug-resistance, relapse and high mortality.

### Resistance to radiation therapy

Ionizing radiation (IR) like X-rays and γ-rays are cost-effective and one of the most extensively used treatments for cancer as a single-modality therapy or combined with surgery and chemotherapy; it is used extensively also for disease management in cured cancer patients^[171]^. IR treatment stalls tumor growth by inducing either apoptosis, necrosis, necroptosis, mitotic catastrophe, autophagy or senescence by inflicting extensive DNA damage in the form of single and double strand breaks, DNA-protein crosslinking and transiently increasing the levels of cytotoxic reactive oxygen species (ROS)^[171,172]^. Although IR is useful for cessation of tumor growth in case of laryngeal, nasopharyngeal, skin, cervical, head-and-neck, prostate and breast cancer, radiotherapy is less or ineffective against bladder cancer, glioblastoma, and soft-tissue cancers, as well as advanced non-small cell lung cancer (NSCLC) due to their intrinsic resistance to IR. Even in the cancers that respond to IR, rapid recurrence as well as acquired resistance to IR is common^[172-174]^. Multiple mechanisms of developing resistance to radiotherapy are known and include: (1) adaptation to radiation via increased anti-oxidant enzymes like SOD, Rel/NF-κB activation, and survivin mediated apoptosis inhibition; (2) intense DNA damage repair by non-homologous end joining (NHEJ) and homologous recombination (HR) via DNA-PK, RAD51, ATM, ATR, and PARP; (3) inflammatory cytokine (IL6, IL1b, IL8) release from tumor and tumor infiltrating lymphocytes (TILs) which in turn increase tumor invasion and successful metastasis; (4) increased cell adhesion to ECM via ICAM and VCAM; (5) activation of hypoxia inducible factor 1a (HIF1a) and HIF1a mediated pro-angiogenic stimuli through VEGF and pro-vasculogenic stimuli through CXCL12; and (6) fibrosis of the tumor microenvironment and immune cell death allowing immune tolerance and tumor invasion^[173-175]^. GH alone, as well as its primary effector IGF1 have a significant effect on IR resistance and post-IR recovery^[176,177]^.

Adult male Wistar rats treated with GH for 7 days, showed an improved rescue from an abdominal mucosal lesion (enteritis) caused by a lethal IR dose^[178]^. Recombinant hGH treatment also rescued irradiated peripheral blood lymphocytes from cell death via Bcl2 activation and restored their cytokine secretory profile^[179]^. BDIX rats with colon tumor xenografts were treated with IR and GH and exhibited a GH-induced decrease in apoptosis and preferential protection in non-tumor intestinal cells and not the irradiated tumor^[180,181]^. The anti-apoptotic effects of GH were validated in irradiated BALB/c mice treated for 35 or 5 days post-irradiation, where recombinant GH treatment significantly restored hematologic and immune recovery compared to saline-treated irradiated mice^[182]^. Non-human primates exhibited similar protective effects of GH^[182]^. IGF1 exhibited an identical anti-apoptotic effect on irradiated BALB/c mice indicating that part of the radioprotection of GH might be mediated through IGF1^[183]^. The studies which used a post-irradiation GH treatment to effect recovery of non-tumor cells, did not include a long-term follow up on any subsequent neoplasmic occurrences in the same patients. This could be pivotal based on the above-mentioned recent reports by Chesnokova *et al*.^[97]^, where a GH excess although anti-apoptotic, actually inhibits DDR, thereby allowing oncogenic transformation in epithelial cells^[97]^.

The effect of GH on cancer cells have reflected the observations with non-tumor cells. Studies by Lobie, Perry, and colleagues have clearly shown that GH does confer radioresistance in tumor cells. GH treated breast cancer cells MDA-MB-435S and T47D, as well as endometrial cancer cell RL95-2 showed markedly reduced DNA damage as well as heightened clonogenic survival post-irradiation^[177]^. GHR-expressing human colorectal cancer cells HCT-8, pretreated with different doses of recombinant hGH, showed a dose-dependent increase in post-irradiation survival while comet assays exhibited reduced DNA damage^[184]^. The effects were again suppressed on exposure to an anti-GHR antibody^[184]^. In animal studies, immunodeficient NIH-III mice with RL95-2 xenografts, when gamma irradiated with or without 100 mg/kg pegvisomant injections every alternate day, showed reduced growth and anti-vascular effects in animals subjected to GHR antagonism^[185]^. Another study involving human cancer patients, looked at pre-operative biopsy and post-irradiation specimens in 98 patients of rectal cancer and found that increased GHR expression was associated with poor response to IR treatment and postulated that GHR-antagonism can actually improve rectal cancer sensitivity to IR therapy^[186]^. Collectively these results bolster the fact that GH action is protective against radiotherapy in human cancers and that functional GH antagonism using a GHR-antagonist helps to sensitize the cancer to IR treatment.

## Mechanisms overlapping GH-action and cancer therapy resistance

ECM remodeling: Composed of about 300 proteins including collagens, proteoglycans and glycoproteins, the ECM is a non-cellular highly dynamic essential support structure within tissues^[187]^. The ECM undergoes remodeling in the form of synthesis, assembly, degradation, reassembly, and chemical modification. Dysregulation in ECM remodeling leads to pathological states and exacerbates disease progression like in cancer^[187]^. Increase in type-IV collagen mediated signaling drastically increased liver metastases in multiple tumor types, especially the ones with markedly higher IGF1R expression^[188]^. In melanoma, therapeutic intervention with BRAF-V600E targeted vemurafenib (PLX4032), increased collagen synthesis *in vitro* and increased collagen deposition *in vivo*^[189]^. Although collagen deposition seems to be beneficial in restricting metastasis of the tumor, increased collagen correlates with increased angiogenic (VEGF) and inflammatory factors (TGFβ)^[190]^. In mice with orthotopic human breast cancer xenografts, metastatic cells in the lymph nodes increased collagen-I density compared to non-metastatic xenografts^[191]^. Collagen-I is now known to cause a metastatic reactivation through a non-canonical collagen receptor tyrosine kinase discoidin domain-containing receptor 1 (DDR1) signaling pathway mediated by JAK2^[192]^. Further up to 50-fold upregulation in expression of a number of collagen genes were found to be associated with drug resistance in ovarian cancer cells^[193]^. Active degradation of collagens and elastins by matrix metalloproteases (MMPs) release angiogenic signals like VEGF and activate immunomodulatory and apoptotic cytokines like TGFβ which help in depolarization of tumor cells and initiation of EMT^[187,190]^. These are hallmarks of early metastasis of an aggressively growing tumor. Therefore, increased collagen deposition as well as increased collagen breakdown are fundamental methods of ECM remodeling^[190]^. GH is known to upregulate both collagen synthesis in human subjects^[194]^ and to increase expression of collagen degrading and TGF-activating MMPs^[107,133]^ in tumors. Additionally, autocrine GH expressing breast cancer cells show increased blood and lymphatic microvessel infiltration in tumor xenografts *in vivo*, due to elevated VEGF signaling^[87]^. No studies have examined the direct relationship of collagen deposition, degradation, and therapy resistance from a GH context, although peripheral GH action can have profound influence around the tumor microenvironment of GHR-positive tumors as well as GHR-expressing normal cells in the immediate milieu. Future findings in this regard can be valuable from the clinical viewpoint of fibrosis and cancer.

MicroRNA mediated epigenetic mechanisms: Broadly conserved across species, microRNAs (miRNA) are a family of single-stranded non-coding RNA sequence 20-25 nucleotides in length located in intronic as well as exonic transcription regions. They regulate almost half of all protein expressions at the post-transcriptional level by direct base-pairing with the 3’-untranslated regions (3’UTR) of corresponding target mRNA and blocking translation^[195]^. Using microarray profiling of MCF-7 breast cancer cells expressing autocrine GH (MCF7-hGH) Zhu, Lobie and colleagues have identified the miRNA cluster 96-182-183 to be under GH regulation^[196]^. The miR-96-182-183 cluster strongly promoted tumor cell migration, invasion and EMT by directly targeting BRMS1L via a STAT3 and STAT5 dependent pathway and promoted distant metastasis of primary mammary tumor in mouse xenografts^[195]^. Interestingly, GHR was one of the targets of miR-96. The miR-96-182-183 cluster have been found to be a critical in tumor proliferation, invasion, and metastases^[197]^. Additionally, another set of sexually dimorphic miRNA expression under GH-mediated JAK2-STAT5 regulation was reported in adult mouse liver. In males miR-1948-5p was expressed and repressed female-biased mRNAs; while miR-802-5p was expressed in female livers and repressed male-biased mRNAs^[198]^. In relevance, while there is no available information yet on miR-1948 in ovarian or endometrial cancers, the miR-802 was found to be a potent onco-suppressor by attenuating EMT via targeting Flotillin2 (Flot2) in prostate cancer^[199]^. Therefore, targeting GHR action as well as identifying sexually dimorphic GH-regulated miRNAs can be a promising drug discovery exercise especially in relevant cancers.

Drug metabolism: The cytochrome-P450 (CYP) family of enzymes can metabolize a broad range of chemotherapy drugs including doxorubicin, CTX, and others and therefore play critical roles in cancer drug resistance^[200]^. The cytochrome P450 family of proteins located in the inner mitochondrial membrane or endoplasmic reticulum of the cells are the major oxidizing enzymes of the electron transport chain. The CYPs are broadly divided into xenobiotic (CYP1-4) and endogenous (CYP7-51). They have been implicated strongly as drivers of several different types of human cancers and have been implicated in primary, malignant, and metastatic stages of the disease^[201]^. Several classes of CYP-inhibitors have been or are under development for the treatment of prostate cancer. In 1995, it was found that pulsatile GH can induce expression of CYP2A2 and CYP3A2 in GH-depleted male but not female rats^[202]^. Multiple studies have reported modulation of hepatic CYPs (CYP2D6, CYP3A) due to GH treatment in GH-deficient human subjects^[203,204]^. Currently there are no direct query into the direct relationship of GH-GHR axis and variations in CYP expression or activity, during drug-treatment in cancer.

In addition to the above, an important method of drug metabolism in human patients is caused by the gut microbiome^[205]^. Increasing volume of research has started to make researchers cognizant of the role that the gut microbiota exerts in determining the efficacy of several classes of drugs including anti-cancer therapeutics^[206,207]^. The delineation of good versus bad microbiome in the context of different cancers^[208,209]^ is gradually being clarified; while we do not know yet how GH excess, deficit, or resistance affects the gut microbiota^[210]^ and its response in cancer and other disease sets. This unexplored area of research can provide vital clues not only for cancer therapy, but also for metabolic dysfunctions like obesity, insulin resistance, and gastrointestinal pathologies.

## Conclusion

Our current understanding of GH mediated cancer therapy resistance is a function of GHR hyperactivation due to increased autocrine/paracrine as well as endocrine GH. A constitutive activation of GHR is also known, as in the case of the P495T mutation, disabling SOCS2 binding to the activated-GHR^[79-81]^. However, the effect of this or other constitutive GHR activations though of significant interest are yet unknown. The existing body of evidences justify GHR-antagonism as a viable approach as monotherapy in cancer^[211]^. However, the identification of a GH dependence of GHR-positive human tumors in driving a distinct radio- and chemo-resistant phenotype is unique and clinically relevant. Pegvisomant, as an existing example of a GHR-antagonist, can be combined with specific anti-cancer therapeutic approaches to improve treatment efficacy. The collection of the above information does warrant GHR-antagonism as a critical strategy in re-sensitizing tumors resistant to a range of anti-cancer therapies. Hitherto, an appropriately designed clinical trial combining pegvisomant or any agent that inhibits GH action with chemo-or radio- or targeted- or immune-therapy does not exist. In a 2015 report, a 72-year male patient with acromegaly and prior colorectal cancer history was diagnosed with breast cancer^[212]^. Following no response from pituitary surgery or SST-analog treatments, he was put on pegvisomant which successfully normalized his IGF1 levels. Following breast cancer surgery, the patient discontinued pegvisomant, contrary to medical advice. He was subsequently detected with two pulmonary metastases and elevated serum IGF1 and was put back on pegvisomant and tamoxifen. In 4-months IGF1 normalized again, the metastatic lesion in left lung reduced, and a 24-month follow-up showed further reduction in the secondary tumor and a stabilized metastases^[212]^. Currently there is a significant pharmaceutical interest in attenuating GH action as is evident from recent strategies in development or in trial including SSTR agonists, dopamine agonists, GH analogs, antisense oligonucleotides, anti-GHR as well as anti-IGF1R monoclonal antibodies, and small molecules aiming at intercepting the GH-GHR mediated signaling^[4]^. While the process of discovery of new therapeutics is an uphill task, re-positioning existing drugs^[213]^ or combining mechanistically relevant drugs^[214]^ has been identified as a more immediate and highly effective solution in tackling the need for millions of patients worldwide^[215]^. The scientific rationale for combining GHR-antagonism with existing anti-cancer treatments, that we present in this review, appear to be viable and systematic *in vivo* studies specifically validating this approach should pave the way for a clinical trial in immediate future.

## Declarations

### Copyright

© The Author(s) 2019.

## References

[1] Peters GJ (2018). Cancer drug resistance: a new perspective.. Cancer Drug Resist.

[2] Basu R, Qian Y, Kopchick JJ (2018). Mechanisms in endocrinology: lessons from growth hormone receptor gene disrupted mice: are there benefits of endocrine defects?. Eur J Endocrinol.

[3] Kopchick JJ, List EO, Kelder B, Gosney ES, Berryman DE (2014). Evaluation of growth hormone (GH) action in mice: discovery of GH receptor antagonists and clinical indications.. Mol Cell Endocrinol.

[4] Lu M, Flanagan JU, Langley RJ, Hay MP, Perry JK (2019). Targeting growth hormone function: strategies and therapeutic applications.. Signal Transduct Target Ther.

[5] http://linkinghub.elsevier.com/retrieve/pii/S1877117315002161.

[6] Pombo M, Pombo CM, Garcia A, Caminos E, Gualillo O (2001). Hormonal control of growth hormone secretion.. Horm Res..

[7] Khatib N, Gaidhane S, Gaidhane AM, Khatib M, Simkhada P (2014). Ghrelin: ghrelin as a regulatory Peptide in growth hormone secretion.. J Clin Diagn Res.

[8] Dimaraki EV, Jaffe CA (2006). Role of endogenous ghrelin in growth hormone secretion, appetite regulation and metabolism.. Rev Endocr Metab Disord.

[9] Müller EE (1970). Brain catecholamines and growth hormone release. Aspects of Neuroendocrinology..

[10] Buonomo FC, Zimmermann NG, Lauterio TJ, Scanes CG (1984). Catecholamine involvement in the control of growth hormone secretion in the domestic fowl.. Gen Comp Endocrinol..

[11] Chang JP, Marchant TA, Cook AF, Nahorniak CS, Peter RE (1985). Influences of catecholamines on growth hormone release in female goldfish, Carassius auratus.. Neuroendocrinology.

[12] Pritzlaff CJ, Wideman L, Blumer J, Jensen M, Abbott RD (2000). Catecholamine release, growth hormone secretion, and energy expenditure during exercise vs. recovery in men.. J Appl Physiol.

[13] Brooks AJ, Waters MJ (2010). The growth hormone receptor: mechanism of activation and clinical implications.. Nat Rev Endocrinol.

[14] Waters MJ, Brooks AJ (2015). JAK2 activation by growth hormone and other cytokines.. Biochem J.

[15] Rowland JE, Lichanska AM, Kerr LM, White M, D’Aniello EM (2005). In vivo analysis of growth hormone receptor signaling domains and their associated transcripts.. Mol Cell Biol.

[16] Carter-Su C, King AP, Argetsinger LS, Smit LS, Vanderkuur J (1996). Signalling pathway of GH.. Endocr J.

[17] Jin H, Lanning NJ, Carter-Su C (2008). JAK2, but not Src family kinases, is required for STAT, ERK, and Akt signaling in response to growth hormone in preadipocytes and hepatoma cells.. Mol Endocrinol.

[18] Bocharov EV, Lesovoy DM, Bocharova OV, Urban AS, Pavlov KV (2018). Structural basis of the signal transduction via transmembrane domain of the human growth hormone receptor.. Biochim Biophys acta Gen Subj.

[19] Zhu T, Goh EL, Graichen R, Ling L, Lobie PE (2001). Signal transduction via the growth hormone receptor.. Cell Signal.

[20] Keene DE, Suescun MO, Bostwick MG, Chandrashekar V, Bartke A (2015). Puberty is delayed in male growth hormone receptor gene-disrupted mice.. J Androl.

[21] Bougnères PF (1993). Growth hormone effects on carbohydrate and lipid metabolism in childhood.. Horm Res.

[22] DAVIDSON MB (1987). Effect of growth hormone on carbohydrate and lipid metabolism.. Endocr Rev.

[23] Møller N, Jørgensen JOL (2009). Effects of growth hormone on glucose, lipid, and protein metabolism in human subjects.. Endocr Rev.

[24] Vijayakumar A, Novosyadlyy R, Wu Y, Yakar S, LeRoith D (2010). Biological effects of growth hormone on carbohydrate and lipid metabolism.. Growth Horm IGF Res.

[25] Duran-Ortiz S, Berryman DE, Kopchick JJ (2019). Growth hormone impact on adipose tissue and aging.. Curr Opin Endocr Metab Res.

[26] Berryman D, List E (2017). Growth Hormone’s effect on adipose tissue: quality versus quantity.. Int J Mol Sci.

[27] Duran-Ortiz S, Brittain AL, Kopchick JJ (2017). The impact of growth hormone on proteomic profiles: a review of mouse and adult human studies.. Clin Proteomics.

[28] Bartke A (2011). Pleiotropic effects of growth hormone signaling in aging.. Trends Endocrinol Metab.

[29] Ashpole NM, Logan S, Yabluchanskiy A, Mitschelen MC, Yan H (2017). IGF-1 has sexually dimorphic, pleiotropic, and time-dependent effects on healthspan, pathology, and lifespan.. GeroScience.

[30] Sonntag WE, Lynch CD, Cefalu WT, Ingram RL, Bennett SA (1999). Pleiotropic effects of growth hormone and insulin-like growth factor (IGF)-1 on biological aging: inferences from moderate caloric-restricted animals.. J Gerontol A Biol Sci Med Sci.

[31] Basu A, McFarlane HG, Kopchick JJ (2017). Spatial learning and memory in male mice with altered growth hormone action.. Horm Behav.

[32] Gosney ES, Jara A, Basu A, Kopchick JJ (2012). GH in the central nervous system: lessons from the growth hormone receptor knockout mouse..

[33] Guevara-Aguirre J, Rosenbloom AL, Balasubramanian P, Teran E, Guevara-Aguirre M (2015). GH receptor deficiency in ecuadorian adults is associated with obesity and enhanced insulin sensitivity.. J Clin Endocrinol Metab.

[34] Laron Z (2015). Lessons from 50 years of study of Laron syndrome.. Endocr Pract.

[35] Treadway MT, Zald DH (2011). Reconsidering anhedonia in depression: lessons from translational neuroscience.. Neurosci Biobehav Rev.

[36] Savage MO, Burren CP, Rosenfeld RG (2009). The continuum of growth hormone-IGF-I axis defects causing short stature: diagnostic and therapeutic challenges.. Clin Endocrinol (Oxf).

[37] Laron Z (2004). Laron syndrome (primary growth hormone resistance or insensitivity): the personal experience 1958-2003.. J Clin Endocrinol Metab.

[38] Ginsberg S, Laron Z, Bed MA, Vaisman N (2009). The obesity of patients with Laron syndrome is not associated with excessive nutritional intake.. Obes Res Clin Pract.

[39] Guevara-Aguirre J, Procel P, Guevara C, Guevara-Aguirre M, Rosado V (2016). Despite higher body fat content, Ecuadorian subjects with Laron syndrome have less insulin resistance and lower incidence of diabetes than their relatives.. Growth Horm IGF Res.

[40] Guevara-Aguirre J, Rosenbloom AL, Fielder PJ, Diamond FB, Rosenfeld RG (1993). Growth hormone receptor deficiency in Ecuador: clinical and biochemical phenotype in two populations.. J Clin Endocrinol Metab.

[41] Junnila RK, Duran-Ortiz S, Suer O, Sustarsic EG, Berryman DE (2016). Disruption of the GH receptor gene in adult mice increases maximal lifespan in females.. Endocrinology.

[42] Junnila RK, List EO, Berryman DE, Murrey JW, Kopchick JJ (2013). The GH/IGF-1 axis in ageing and longevity.. Nat Rev Endocrinol.

[43] Krzisnik C, Kolacio Z, Battelino T, Brown M, Parks JS (1999). The “Little People” of the Island of Krk - revisited. Etiology of Hypopituitarism Revealed.. Int J Disabil Hum Dev.

[44] Krzisnik C, Grgurić S, Cvijović K, Laron Z (2010). Longevity of the hypopituitary patients from the island Krk: a follow-up study.. Pediatr Endocrinol Rev.

[45] Salvatori R, Hayashida CY, Aguiar-Oliveira MH, Phillips JA, Souza AHO (1999). Familial Dwarfism due to a novel mutation of the growth hormone-releasing hormone receptor gene 1.. J Clin Endocrinol Metab.

[46] Costa UMM, Oliveira CRP, Salvatori R, Barreto-Filho JAS, Campos VC (2016). Brazilian adult individuals with untreated isolated GH deficiency do not have accelerated subclinical atherosclerosis.. Endocr Connect.

[47] Baumann G, Maheshwari H (1997). The dwarfs of Sindh: severe growth hormone (GH) deficiency caused by a mutation in the GH-releasing hormone receptor gene.. Acta Paediatr Suppl.

[48] Besson A, Salemi S, Gallati S, Jenal A, Horn R (2003). Reduced longevity in untreated patients with isolated growth hormone deficiency.. J Clin Endocrinol Metab.

[49] Rozzi FVR, Koudou Y, Froment A, Le Bouc Y, Botton J (2015). Growth pattern from birth to adulthood in African pygmies of known age.. Nat Commun.

[50] Melmed S (2009). Acromegaly pathogenesis and treatment.. J Clin Invest.

[51] Adelman D, Liebert, Nachtigall, Lamerson, Bakker B (2013). Acromegaly: the disease, its impact on patients, and managing the burden of long-term treatment.. Int J Gen Med.

[52] Dal J, Leisner MZ, Hermansen K, Farkas DK, Bengtsen M (2018). Cancer incidence in patients with acromegaly: a cohort study and meta-analysis of the literature.. J Clin Endocrinol Metab.

[53] Wolinski K, Stangierski A, Dyrda K, Nowicka K, Pelka M (2017). Risk of malignant neoplasms in acromegaly: a case-control study.. J Endocrinol Invest.

[54] Ruchała M, Szczepanek-Parulska E, Fularz M, Woliński K (2012). Risk of neoplasms in acromegaly.. Contemp Oncol (Poznan, Poland).

[55] Wolinski K, Czarnywojtek A, Ruchala M (2014). Risk of thyroid nodular disease and thyroid cancer in patients with acromegaly - meta-analysis and systematic review.. PLoS One.

[56] Cummings EA, Sochett EB, Dekker MG, Lawson ML, Daneman D (1998). Contribution of growth hormone and IGF-I to early diabetic nephropathy in type 1 diabetes.. Diabetes.

[57] Landau D, Israel E, Rivkis I, Kachko L, Schrijvers BF (2003). The effect of growth hormone on the development of diabetic kidney disease in rats.. Nephrol Dial Transplant.

[58] Kumar PA, Brosius FC, Menon RK (2011). The glomerular podocyte as a target of growth hormone action: implications for the pathogenesis of diabetic nephropathy.. Curr Diabetes Rev.

[59] Dekkers OM, Biermasz NR, Pereira AM, Romijn JA, Vandenbroucke JP (2008). Mortality in acromegaly: a metaanalysis.. J Clin Endocrinol Metab.

[60] Ratajczak MZ, Bartke A, Darzynkiewicz Z (2017). Prolonged growth hormone/insulin/insulin-like growth factor nutrient response signaling pathway as a silent killer of stem cells and a culprit in aging.. Stem Cell Rev Reports.

[61] Palmer AJ, Chung MY, List EO, Walker J, Okada S (2009). Age-related changes in body composition of bovine growth hormone transgenic mice.. Endocrinology.

[62] Kucia M, Masternak M, Liu R, Shin DM, Ratajczak J (2013). The negative effect of prolonged somatotrophic/insulin signaling on an adult bone marrow-residing population of pluripotent very small embryonic-like stem cells (VSELs).. Age (Omaha).

[63] Janecka A, Kołodziej-Rzepa M, Biesaga B (2016). Clinical and molecular features of laron syndrome, a genetic disorder protecting from cancer.. In Vivo.

[64] Di Bella G, Colori B, Scanferlato R (2018). The over-expression of GH/GHR in tumour tissues with respect to healthy ones confirms its oncogenic role and the consequent oncosuppressor role of its physiological inhibitor, somatostatin: a review of the literature.. Neuro Endocrinol Lett.

[65] Brittain AL, Basu R, Qian Y, Kopchick JJ (2017). Growth hormone and the epithelial-to-mesenchymal transition.. J Clin Endocrinol Metab.

[66] Kong X, Wu W, Yuan Y, Pandey V, Wu Z (2016). Human growth hormone and human prolactin function as autocrine/paracrine promoters of progression of hepatocellular carcinoma.. Oncotarget.

[67] Chhabra Y, Waters MJ, Brooks AJ (2011). Role of the growth hormone-IGF-1 axis in cancer.. Expert Rev Endocrinol Metab.

[68] Perry JK, Liu DX, Wu ZS, Zhu T, Lobie PE (2013). Growth hormone and cancer: an update on progress.. Curr Opin Endocrinol Diabetes Obes.

[69] Boguszewski CL, Boguszewski MCDS (2019). Growth Hormone’s links to cancer.. Endocr Rev.

[70] Boguszewski CL (2017). Update on GH therapy in adults.. F1000Research.

[71] Poidvin A, Carel JC, Ecosse E, Levy D, Michon J (2018). Increased risk of bone tumors after growth hormone treatment in childhood: a population-based cohort study in France.. Cancer Med.

[72] Swerdlow AJ, Cooke R, Beckers D, Butler G, Carel JC (2019). Risk of meningioma in european patients treated with growth hormone in childhood: results from the SAGhE cohort.. J Clin Endocrinol Metab.

[73] Swerdlow AJ, Cooke R, Beckers D, Borgström B, Butler G (2017). Cancer risks in patients treated with growth hormone in childhood: the SAGhE European cohort study.. J Clin Endocrinol Metab.

[74] Jørgensen JOL, Juul A (2018). Therapy of endocrine disease: growth hormone replacement therapy in adults: 30 years of personal clinical experience.. Eur J Endocrinol.

[75] Höybye C, Christiansen JS (2015). Growth hormone replacement in adults - current standards and new perspectives.. Best Pract Res Clin Endocrinol Metab.

[76] Quigley CA, Child CJ, Zimmermann AG, Rosenfeld RG, Robison LL (2017). Mortality in children receiving growth hormone treatment of growth disorders: data from the genetics and neuroendocrinology of short stature international study.. J Clin Endocrinol Metab.

[77] Child CJ, Zimmermann AG, Chrousos GP, Cummings E, Deal CL (2019). Safety outcomes during pediatric GH therapy: final results from the prospective GeNeSIS observational program.. J Clin Endocrinol Metab.

[78] Terzolo M, Reimondo G, Berchialla P, Ferrante E, Malchiodi E (2017). Acromegaly is associated with increased cancer risk: a survey in Italy.. Endocr Relat Cancer.

[79] Rudd MF (2006). Variants in the GH-IGF axis confer susceptibilityto lung cancer.. Genome Res.

[80] Chhabra Y, Wong HY, Nikolajsen LF, Steinocher H, Papadopulos A (2018). A growth hormone receptor SNP promotes lung cancer by impairment of SOCS2-mediated degradation.. Oncogene.

[81] Cao G, Lu H, Feng J, Shu J, Zheng D (2008). Lung cancer risk associated with Thr495Pro polymorphism of GHR in Chinese population.. Jpn J Clin Oncol.

[82] Sobrier ML, Dastot F, Duquesnoy P, Kandemir N, Yordam N (1997). Nine novel growth hormone receptor gene mutations in patients with laron syndrome.. J Clin Endocrinol Metab.

[83] Laron Z, Kauli R, Lapkina L, Werner H (2017). IGF-I deficiency, longevity and cancer protection of patients with Laron syndrome.. Mutat Res Mutat Res.

[84] Lapkina-Gendler L, Rotem I, Pasmanik-Chor M, Gurwitz D, Sarfstein R (2016). Identification of signaling pathways associated with cancer protection in Laron syndrome.. Endocr Relat Cancer.

[85] Steuerman R, Shevah O, Laron Z (2011). Congenital IGF1 deficiency tends to confer protection against post-natal development of malignancies.. Eur J Endocrinol.

[86] Pollak M, Blouin M, Zhang J, Kopchick JJ (2001). Reduced mammary gland carcinogenesis in transgenic mice expressing a growth hormone antagonist.. Br J Cancer.

[87] Brunet-Dunand SE, Vouyovitch C, Araneda S, Pandey V, Vidal LJP (2009). Autocrine human growth hormone promotes tumor angiogenesis in mammary carcinoma.. Endocrinology.

[88] Perry JK, Wu ZS, Mertani HC, Zhu T, Lobie PE (2017). Tumour-derived human growth hormone as a therapeutic target in oncology.. Trends Endocrinol Metab.

[89] Weroha SJ, Haluska P (2012). The insulin-like growth factor system in cancer.. Endocrinol Metab Clin North Am.

[90] Yuan J, Yin Z, Tao K, Wang G, Gao J (2018). Function of insulin-like growth factor 1 receptor in cancer resistance to chemotherapy.. Oncol Lett.

[91] Kawa MP, Stecewicz I, Piecyk K, Paczkowska E, Rogińska D (2017). The impact of growth hormone therapy on the apoptosis assessment in CD34+ hematopoietic cells from children with growth hormone deficiency.. Int J Mol Sci.

[92] Kastan MB, Canman CE, Leonard CJ (1995). P53, cell cycle control and apoptosis: implications for cancer.. Cancer Metastasis Rev.

[93] Chen J (2016). The cell-cycle arrest and apoptotic functions of p53 in tumor initiation and progression.. Cold Spring Harb Perspect Med.

[94] van Oijen MG, Slootweg PJ (2000). Gain-of-function mutations in the tumor suppressor gene p53.. Clin Cancer Res.

[95] Arnold RE, Weigent DA (2004). The inhibition of apoptosis in EL4 lymphoma cells overexpressing growth hormone.. Neuroimmunomodulation.

[96] Chesnokova V, Zonis S, Zhou C, Recouvreux MV, Ben-Shlomo A (2016). Growth hormone is permissive for neoplastic colon growth.. Proc Natl Acad Sci U S A.

[97] Chesnokova V, Zonis S, Barrett R, Kameda H, Wawrowsky K (2019). Excess growth hormone suppresses DNA damage repair in epithelial cells.. JCI insight.

[98] Chesnokova V, Zhou C, Ben-Shlomo A, Zonis S, Tani Y (2013). Growth hormone is a cellular senescence target in pituitary and nonpituitary cells.. Proc Natl Acad Sci U S A.

[99] Waters MJ, Conway-Campbell BL (2004). The oncogenic potential of autocrine human growth hormone in breast cancer.. Proc Natl Acad Sci U S A.

[100] Conway-Campbell BL, Wooh JW, Brooks AJ, Gordon D, Brown RJ (2007). Nuclear targeting of the growth hormone receptor results in dysregulation of cell proliferation and tumorigenesis.. Proc Natl Acad Sci U S A.

[101] Cheng Q, Chen J (2010). Mechanism of p53 stabilization by ATM after DNA damage.. Cell Cycle.

[102] Podlutsky A, Valcarcel-Ares MN, Yancey K, Podlutskaya V, Nagykaldi E (2017). The GH/IGF-1 axis in a critical period early in life determines cellular DNA repair capacity by altering transcriptional regulation of DNA repair-related genes: implications for the developmental origins of cancer.. GeroScience.

[103] Minoia M, Gentilin E, Molè D, Rossi M, Filieri C (2012). Growth hormone receptor blockade inhibits growth hormone-induced chemoresistance by restoring cytotoxic-induced apoptosis in breast cancer cells independently of estrogen receptor expression.. J Clin Endocrinol Metab.

[104] Zatelli MC, Minoia M, Molè D, Cason V, Tagliati F (2009). Growth hormone excess promotes breast cancer chemoresistance.. J Clin Endocrinol Metab.

[105] Gentilin E, Minoia M, Bondanelli M, Tagliati F, Degli Uberti EC (2017). Growth hormone differentially modulates chemoresistance in human endometrial adenocarcinoma cell lines.. Endocrine.

[106] Bogazzi F, Ultimieri F, Raggi F, Russo D, Vanacore R (2004). Growth hormone inhibits apoptosis in human colonic cancer cell lines: antagonistic effects of peroxisome proliferator activated receptor-γ ligands.. Endocrinology.

[107] Subramani R, Lopez-Valdez R, Salcido A, Boopalan T, Arumugam A (2014). Growth hormone receptor inhibition decreases the growth and metastasis of pancreatic ductal adenocarcinoma.. Exp Mol Med.

[108] Subramani R, Nandy SB, Pedroza DA, Lakshmanaswamy R (2017). Role of growth hormone in breast cancer.. Endocrinology.

[109] Kaulsay KK, Mertani HC, Törnell J, Morel G, Lee KO (1999). Autocrine stimulation of human mammary carcinoma cell proliferation by human growth hormone.. Exp Cell Res.

[110] Mertani HC, Zhu T, Goh EL, Lee KO, Morel G (2001). Autocrine human growth hormone (hGH) regulation of human mammary carcinoma cell gene expression. Identification of CHOP as a mediator of hGH-stimulated human mammary carcinoma cell survival.. J Biol Chem.

[111] Kalluri R, Weinberg RA (2009). The basics of epithelial-mesenchymal transition.. J Clin Invest.

[112] Lamouille S, Xu J, Derynck R (2014). Molecular mechanisms of epithelial-mesenchymal transition.. Nat Rev Mol Cell Biol.

[113] Singh A, Settleman J (2010). EMT, cancer stem cells and drug resistance: an emerging axis of evil in the war on cancer.. Oncogene.

[114] Du B, Shim J (2016). Targeting epithelial-mesenchymal transition (EMT) to overcome drug resistance in cancer.. Molecules.

[115] Tsai JH, Yang J (2013). Epithelial-mesenchymal plasticity in carcinoma metastasis.. Genes Dev.

[116] Mitra A, Mishra L, Li S (2015). EMT, CTCs and CSCs in tumor relapse and drug-resistance.. Oncotarget.

[117] Kong D, Li Y, Wang Z, Sarkar F (2011). Cancer stem cells and epithelial-to-mesenchymal transition (EMT)-phenotypic cells: are they cousins or twins?. Cancers (Basel).

[118] Aiello NM, Brabletz T, Kang Y, Nieto MA, Weinberg RA (2017). Upholding a role for EMT in pancreatic cancer metastasis.. Nature.

[119] Fischer KR, Durrans A, Lee S, Sheng J, Li F (2015). Epithelial-to-mesenchymal transition is not required for lung metastasis but contributes to chemoresistance.. Nature.

[120] Kurrey NK, Jalgaonkar SP, Joglekar AV, Ghanate AD, Chaskar PD (2009). Snail and slug mediate radioresistance and chemoresistance by antagonizing p53-mediated apoptosis and acquiring a stem-like phenotype in ovarian cancer cells.. Stem Cells.

[121] Yang H, Zhang G, Che X, Yu S (2018). Slug inhibition increases radiosensitivity of nasopharyngeal carcinoma cell line C666-1.. Exp Ther Med.

[122] Pulkka OP, Nilsson B, Sarlomo-Rikala M, Reichardt P, Eriksson M (2017). SLUG transcription factor: a pro-survival and prognostic factor in gastrointestinal stromal tumour.. Br J Cancer.

[123] Yochum ZA, Socinski MA, Burns TF (2016). Paradoxical functions of ZEB1 in EGFR-mutant lung cancer: tumor suppressor and driver of therapeutic resistance.. J Thorac Dis.

[124] Yochum ZA, Cades J, Wang H, Chatterjee S, Simons BW (2019). Targeting the EMT transcription factor TWIST1 overcomes resistance to EGFR inhibitors in EGFR-mutant non-small-cell lung cancer.. Oncogene.

[125] Mukhina S, Mertani HC, Guo K, Lee KO, Gluckman PD (2004). Phenotypic conversion of human mammary carcinoma cells by autocrine human growth hormone.. Proc Natl Acad Sci U S A.

[126] Yilmaz M, Christofori G (2009). EMT, the cytoskeleton, and cancer cell invasion.. Cancer Metastasis Rev.

[127] Aiello NM, Maddipati R, Norgard RJ, Balli D, Li J (2018). EMT subtype influences epithelial plasticity and mode of cell migration.. Dev Cell.

[128] Zhang X, Zhu T, Chen Y, Mertani HC, Lee KO (2003). Human growth hormone-regulated HOXA1 is a human mammary epithelial oncogene.. J Biol Chem.

[129] Zhu T, Starling-Emerald B, Zhang X, Lee KO, Gluckman PD (2005). Oncogenic transformation of human mammary epithelial cells by autocrine human growth hormone.. Cancer Res.

[130] Shafiei F, Rahnama F, Pawella L, Mitchell MD, Gluckman PD (2008). DNMT3A and DNMT3B mediate autocrine hGH repression of plakoglobin gene transcription and consequent phenotypic conversion of mammary carcinoma cells.. Oncogene.

[131] Wang JJ, Chong QY, Sun XB, You ML, Pandey V (2017). Autocrine hGH stimulates oncogenicity, epithelial-mesenchymal transition and cancer stem cell-like behavior in human colorectal carcinoma.. Oncotarget.

[132] Basu R, Wu S, Kopchick J (2017). Targeting growth hormone receptor in human melanoma cells attenuates tumor progression and epithelial mesenchymal transition via suppression of multiple oncogenic pathways.. Oncotarget.

[133] Pandey V, Perry JK, Mohankumar KM, Kong XJ, Liu SM (2008). Autocrine human growth hormone stimulates oncogenicity of endometrial carcinoma cells.. Endocrinology.

[134] Chen Z, Shi T, Zhang L, Zhu P, Deng M (2016). Mammalian drug efflux transporters of the ATP binding cassette (ABC) family in multidrug resistance: a review of the past decade.. Cancer Lett.

[135] Montanari F, Ecker GF (2015). Prediction of drug-ABC-transporter interaction - recent advances and future challenges.. Adv Drug Deliv Rev.

[136] Heimerl S, Bosserhoff AK, Langmann T, Ecker J, Schmitz G (2007). Mapping ATP-binding cassette transporter gene expression profiles in melanocytes and melanoma cells.. Melanoma Res.

[137] Robey RW, Pluchino KM, Hall MD, Fojo AT, Bates SE (2018). Revisiting the role of ABC transporters in multidrug-resistant cancer.. Nat Rev Cancer.

[138] Kathawala RJ, Gupta P, Ashby CR, Chen ZS (2015). The modulation of ABC transporter-mediated multidrug resistance in cancer: a review of the past decade.. Drug Resist Updat.

[139] Szakács G, Annereau JP, Lababidi S, Shankavaram U, Arciello A (2004). Predicting drug sensitivity and resistance.. Cancer Cell.

[140] Dvorak P, Pesta M, Soucek P (2017). ABC gene expression profiles have clinical importance and possibly form a new hallmark of cancer.. Tumor Biol.

[141] Choi CH (2005). ABC transporters as multidrug resistance mechanisms and the development of chemosensitizers for their reversal.. Cancer Cell Int.

[142] Ejendal KFK, Hrycyna CA (2002). Multidrug resistance and cancer: the role of the human ABC transporter ABCG2.. Curr Protein Pept Sci.

[143] Jaramillo AC, Al Saig F, Cloos J, Jansen G, Peters GJ (2018). How to overcome ATP-binding cassette drug efflux transporter-mediated drug resistance?. Cancer Drug Resist.

[144] Chen KG, Valencia JC, Gillet JP, Hearing VJ, Gottesman MM (2009). Involvement of ABC transporters in melanogenesis and the development of multidrug resistance of melanoma.. Pigment Cell Melanoma Res.

[145] Chen KG, Valencia JC, Lai B, Zhang G, Paterson JK (2006). Melanosomal sequestration of cytotoxic drugs contributes to the intractability of malignant melanomas.. Proc Natl Acad Sci.

[146] Bougen NM, Yang T, Chen H, Lobie PE, Perry JK (2011). Autocrine human growth hormone reduces mammary and endometrial carcinoma cell sensitivity to mitomycin C.. Oncol Rep.

[147] Holtz AN, Yee D, Beckwith H (2018). Abstract 5839: growth hormone receptor (GHR) expression confers resistance to ruxolitinib in endocrine-resistant breast cancer cells.. Cancer Res.

[148] Basu R, Baumgaertel N, Wu S, Kopchick JJ (2017). Growth hormone receptor knockdown sensitizes human melanoma cells to chemotherapy by attenuating expression of ABC drug efflux pumps.. Horm Cancer.

[149] Arumugam A, Subramani R, Nandy SB, Terreros D, Dwivedi AK (2019). Silencing growth hormone receptor inhibits estrogen receptor negative breast cancer through ATP-binding cassette sub-family G member 2.. Exp Mol Med.

[150] Wu AML, Dalvi P, Lu X, Yang M, Riddick DS (2013). Induction of multidrug resistance transporter ABCG2 by prolactin in human breast cancer cells.. Mol Pharmacol.

[151] Santisteban M (2010). ABC transporters as molecular effectors of pancreatic oncogenic pathways: the hedgehog-GLI model.. J Gastrointest Cancer.

[152] Fletcher JI, Haber M, Henderson MJ, Norris MD (2010). ABC transporters in cancer: more than just drug efflux pumps.. Nat Rev Cancer.

[153] Pahnke J, Langer O, Krohn M (2014). Alzheimer’s and ABC transporters - new opportunities for diagnostics and treatment.. Neurobiol Dis.

[154] Abuznait AH, Kaddoumi A (2012). Role of ABC transporters in the pathogenesis of Alzheimer’s disease.. ACS Chem Neurosci.

[155] Mahringer A, Fricker G (2016). ABC transporters at the blood-brain barrier.. Expert Opin Drug Metab Toxicol.

[156] Tarling EJ, de Aguiar Vallim TQ, Edwards PA (2013). Role of ABC transporters in lipid transport and human disease.. Trends Endocrinol Metab.

[157] Schumacher T, Benndorf RA (2017). ABC transport proteins in cardiovascular disease-a brief summary.. Molecules.

[158] Gottesman MM, Ambudkar SV (2001). Overview: ABC transporters and human disease.. J Bioenerg Biomembr.

[159] Stanton B (2002). ABC transporters in liver, kidney, and intestine.. Kidney Int.

[160] Stefková J, Poledne R, Hubácek JA (2004). ATP-binding cassette (ABC) transporters in human metabolism and diseases.. Physiol Res.

[161] Batlle E, Clevers H (2017). Cancer stem cells revisited.. Nat Med.

[162] Ciurea ME, Georgescu AM, Purcaru SO, Artene SA, Emami GH (2014). Cancer stem cells: biological functions and therapeutically targeting.. Int J Mol Sci.

[163] Phi LTH, Sari IN, Yang YG, Lee SH, Jun N (2018). Cancer stem cells (CSCs) in drug resistance and their therapeutic implications in cancer treatment.. Stem Cells Int.

[164] Florea V, Majid SS, Kanashiro-Takeuchi RM, Cai RZ, Block NL (2014). Agonists of growth hormone-releasing hormone stimulate self-renewal of cardiac stem cells and promote their survival.. Proc Natl Acad Sci.

[165] Sackmann-Sala L, Guidotti JE, Goffin V (2015). Minireview: prolactin regulation of adult stem cells.. Mol Endocrinol.

[166] Sackmann-Sala L, Goffin V (2015). Prolactin-induced prostate tumorigenesis.. Adv Exp Med Biol.

[167] Neradugomma NK, Subramaniam D, Tawfik OW, Goffin V, Kumar TR (2014). Prolactin signaling enhances colon cancer stemness by modulating Notch signaling in a Jak2-STAT3/ERK manner.. Carcinogenesis.

[168] Chen YJ, Zhang X, Wu ZS, Wang JJ, Lau AYC (2015). Autocrine human growth hormone stimulates the tumor initiating capacity and metastasis of estrogen receptor-negative mammary carcinoma cells.. Cancer Lett.

[169] Lombardi S, Honeth G, Ginestier C, Shinomiya I, Marlow R (2014). Growth hormone is secreted by normal breast epithelium upon progesterone stimulation and increases proliferation of stem/progenitor cells.. Stem Cell Reports.

[170] Chen YJ, You ML, Chong QY, Pandey V, Zhuang QS (2017). Autocrine human growth hormone promotes invasive and cancer stem cell-like behavior of hepatocellular carcinoma cells by STAT3 dependent inhibition of CLAUDIN-1 expression.. Int J Mol Sci.

[171] Chen HHW, Kuo MT (2017). Improving radiotherapy in cancer treatment: Promises and challenges.. Oncotarget.

[172] Baskar R, Lee KA, Yeo R, Yeoh KW (2012). Cancer and radiation therapy: current advances and future directions.. Int J Med Sci.

[173] Kim BM, Hong Y, Lee S, Liu P, Lim JH (2015). Therapeutic implications for overcoming radiation resistance in cancer therapy.. Int J Mol Sci.

[174] Barker HE, Paget JTE, Khan AA, Harrington KJ (2015). The tumour microenvironment after radiotherapy: mechanisms of resistance and recurrence.. Nat Rev Cancer.

[175] Willers H, Azzoli CG, Santivasi WL, Xia F (2013). Basic mechanisms of therapeutic resistance to radiation and chemotherapy in lung cancer.. Cancer J.

[176] Redelman D, Welniak LA, Taub D, Murphy WJ Neuroendocrine hormones such as growth hormone and prolactin are integral members of the immunological cytokine network.. Cell Immunol.

[177] Bougen NM, Steiner M, Pertziger M, Banerjee A, Brunet-Dunand SE (2012). Autocrine human GH promotes radioresistance in mammary and endometrial carcinoma cells.. Endocr Relat Cancer.

[178] Prieto I, Gómez de Segura IA, García Grande A, García P, Carralero I (1998). Morphometric and proliferative effects of growth hormone on radiation enteritis in the rat.. Rev Esp Enferm Dig.

[179] Lempereur L, Brambilla D, Maria Scoto G, D’Alcamo M, Goffin V (2003). Growth hormone protects human lymphocytes from irradiation-induced cell death.. Br J Pharmacol.

[180] Morante J, Vallejo-Cremades MT, Gómez-García L, Vázquez I, Gómez-de-Segura IA (2003). Differential action of growth hormone in irradiated tumoral and nontumoral intestinal tissue.. Dig Dis Sci.

[181] Caz V, Elvira M, Tabernero M, Grande AG, Lopez-Plaza B (2015). Growth hormone protects the intestine preserving radiotherapy efficacy on tumors: a short-term study.. PLoS One.

[182] Chen BJ, Deoliveira D, Spasojevic I, Sempowski GD, Jiang C (2010). Growth hormone mitigates against lethal irradiation and enhances hematologic and immune recovery in mice and nonhuman primates.. PLoS One.

[183] Zhou D, Deoliveira D, Kang Y, Choi SS, Li Z (2013). Insulin-like growth factor 1 mitigates hematopoietic toxicity after lethal total body irradiation.. Int J Radiat Oncol Biol Phys.

[184] Wu XY, Chen C, Yao XQ, Cao QH, Xu Z (2014). Growth hormone protects colorectal cancer cells from radiation by improving the ability of DNA damage repair.. Mol Med Rep.

[185] Evans A, Jamieson SMF, Liu DX, Wilson WR, Perry JK (2016). Growth hormone receptor antagonism suppresses tumour regrowth after radiotherapy in an endometrial cancer xenograft model.. Cancer Lett.

[186] Wu X, Wan M, Li G, Xu Z, Chen C (2006). Growth hormone receptor overexpression predicts response of rectal cancers to pre-operative radiotherapy.. Eur J Cancer.

[187] Bonnans C, Chou J, Werb Z (2014). Remodelling the extracellular matrix in development and disease.. Nat Rev Mol Cell Biol.

[188] Burnier J V, Wang N, Michel RP, Hassanain M, Li S (2011). Type IV collagen-initiated signals provide survival and growth cues required for liver metastasis.. Oncogene.

[189] Jenkins MH, Croteau W, Mullins DW, Brinckerhoff CE (2015). The BRAFV600E inhibitor, PLX4032, increases type I collagen synthesis in melanoma cells.. Matrix Biol.

[190] Fang M, Yuan J, Peng C, Li Y (2014). Collagen as a double-edged sword in tumor progression.. Tumor Biol.

[191] Rizwan A, Bulte C, Kalaichelvan A, Cheng M, Krishnamachary B (2015). Metastatic breast cancer cells in lymph nodes increase nodal collagen density.. Sci Rep.

[192] Gao H, Chakraborty G, Zhang Z, Akalay I, Gadiya M (2016). Multi-organ site metastatic reactivation mediated by non-canonical discoidin domain receptor 1 signaling.. Cell.

[193] Januchowski R, Świerczewska M, Sterzyńska K, Wojtowicz K, Nowicki M (2016). Increased expression of several collagen genes is associated with drug resistance in ovarian cancer cell lines.. J Cancer.

[194] Doessing S, Heinemeier KM, Holm L, Mackey AL, Schjerling P (2010). Growth hormone stimulates the collagen synthesis in human tendon and skeletal muscle without affecting myofibrillar protein synthesis.. J Physiol.

[195] An X, Sarmiento C, Tan T, Zhu H (2017). Regulation of multidrug resistance by microRNAs in anti-cancer therapy.. Acta Pharm Sin B.

[196] Zhang W, Qian P, Zhang X, Zhang M, Wang H (2015). Autocrine/paracrine human growth hormone-stimulated microRNA 96-182-183 cluster promotes epithelial-mesenchymal transition and invasion in breast cancer.. J Biol Chem.

[197] Ma Y, Liang AJ, Fan YP, Huang YR, Zhao XM (2016). Dysregulation and functional roles of miR-183-96-182 cluster in cancer cell proliferation, invasion and metastasis.. Oncotarget.

[198] Hao P, Waxman DJ (2018). Functional roles of sex-biased, growth hormone-regulated microRNAs miR-1948 and miR-802 in young adult mouse liver.. Endocrinology.

[199] Wang D, Lu G, Shao Y, Xu D (2017). microRNA-802 inhibits epithelial-mesenchymal transition through targeting flotillin-2 in human prostate cancer.. Biosci Rep.

[200] Rodriguez-Antona C, Ingelman-Sundberg M (2006). Cytochrome P450 pharmacogenetics and cancer.. Oncogene.

[201] Bruno RD, Njar VCO (2007). Targeting cytochrome P450 enzymes: a new approach in anti-cancer drug development.. Bioorg Med Chem.

[202] Waxman DJ, Ram PA, Pampori NA, Shapiro BH (1995). Growth hormone regulation of male-specific rat liver P450s 2A2 and 3A2: induction by intermittent growth hormone pulses in male but not female rats rendered growth hormone deficient by neonatal monosodium glutamate.. Mol Pharmacol.

[203] Cheung NW, Liddle C, Coverdale S, Lou JC, Boyages SC (1996). Growth hormone treatment increases cytochrome P450-mediated antipyrine clearance in man.. J Clin Endocrinol Metab.

[204] Gil Berglund E, Johannsson G, Beck O, Bengtsson BA, Rane A (2002). Growth hormone replacement therapy induces codeine clearance.. Eur J Clin Invest.

[205] Bhatt AP, Redinbo MR, Bultman SJ (2017). The role of the microbiome in cancer development and therapy.. CA Cancer J Clin.

[206] Pouncey AL, Scott AJ, Alexander JL, Marchesi J, Kinross J (2018). Gut microbiota, chemotherapy and the host: the influence of the gut microbiota on cancer treatment.. Ecancermedicalscience.

[207] Fessler JL, Gajewski TF (2017). The microbiota: a new variable impacting cancer treatment outcomes.. Clin Cancer Res.

[208] De Almeida CV, de Camargo MR, Russo E, Amedei A (2018). Role of diet and gut microbiota on colorectal cancer immunomodulation.. World J Gastroenterol.

[209] Rea D, Coppola G, Palma G, Barbieri A, Luciano A (2018). Microbiota effects on cancer: from risks to therapies.. Oncotarget.

[210] Yan J, Charles JF (2018). Gut Microbiota and IGF-1.. Calcif Tissue Int.

[211] Friend KE (2001). Cancer and the potential place for growth hormone receptor antagonist therapy.. Growth Horm IGF Res.

[212] Leporati P, Fonte R, de Martinis L, Zambelli A, Magri F (2015). A male patient with acromegaly and breast cancer: treating acromegaly to control tumor progression.. BMC Cancer.

[213] Telleria CM (2012). Drug repurposing for cancer therapy.. J Cancer Sci Ther.

[214] Langdon CG, Platt JT, Means RE, Iyidogan P, Mamillapalli R (2017). Combinatorial screening of pancreatic adenocarcinoma reveals sensitivity to drug combinations including bromodomain inhibitor plus neddylation inhibitor.. Mol Cancer Ther.

[215] Nosengo N (2016). Can you teach old drugs new tricks?. Nature.

[216] Wu XY, Chen C, Yao XQ, Cao QH, Xu Z (2014). Growth hormone protects colorectal cancer cells from radiation by improving the ability of DNA damage repair.. Mol Med Rep.

